# Efficacy and safety of heparin plus dexamethasone after partial splenic embolization for liver cirrhosis with massive splenomegaly

**DOI:** 10.1186/s12876-022-02580-8

**Published:** 2022-11-19

**Authors:** Haohao Lu, Chuansheng Zheng, Bin Xiong, Xiangwen Xia

**Affiliations:** 1 Department of Radiology, Wuhan Union Hospital, Tongji Medical College, Huazhong University of Science and Technology, Jiefang Avenue #1277, Wuhan, 430022 China; 2 Hubei Province Key Laboratory of Molecular Imaging, Wuhan, 430022 China

**Keywords:** Partial splenic embolization, Complications, Post-embolization syndrome, Portal vein thrombosis, Refractory ascites, Massive splenomegaly, Cirrhosis

## Abstract

**Purpose:**

The aim of this study was to investigate the efficacy and safety of the combination of low-molecular-weight heparin + dexamethasone after partial splenic embolization in cirrhotic patients with massive splenomegaly.

**Methods:**

This study included 116 patients with liver cirrhosis complicated with massive splenomegaly who underwent PSE in Union Hospital from January 2016 to December 2019, and they met the criteria. They were divided into two groups: PSE + Hep + Dex group (N = 54) and PSE group (N = 62). We conducted a retrospective study to analyze the efficacy and safety of the two groups of patients.

**Results:**

The volume of splenic embolization was 622.34 ± 157.06 cm^3^ in the PSE + Hep + DEX group and 587.62 ± 175.33 cm^3^ in the PSE group (*P* = 0.306). There was no statistically difference in the embolization rate of the spleen between the two groups (*P* = 0.573). WBC peaked 1 week after PSE and PLT peaked 1 month after PSE in both groups; it gradually decreased later, but was significantly higher than the preoperative level during the 12-month follow-up period. The incidences of abdominal pain (46.3% vs 66.1%, *P* = 0.039), fever (38.9% vs 75.8%, *P* < 0.001), PVT (1.9% vs 12.9%, *P* = 0.026), refractory ascites (5.6% vs 19.4%, *P* = 0.027) were lower in the PSE + Hep + DEX group than in the PSE group. The VAS score of abdominal pain in PSE group was higher than that in PSE + Hep + DEX group on postoperative days 2–8 (*P* < 0.05). Splenic abscess occurred in 1(1.6%) patient in the PSE group and none (0.0%) in the PSE + Hep + DEX group (*P* = 0.349).

**Conclusions:**

The combined use of dexamethasone and low-molecular-weight heparin after PSE is a safe and effective treatment strategy that can significantly reduce the incidence of complications after PSE (such as post-embolization syndrome, PVT, refractory ascites).

## Introduction

Cirrhosis is a diffuse irreversible damage to the liver resulting from different etiologies and is pathologically characterized by extensive hepatocyte necrosis, regeneration, and pseudolobule formation [1, 2]. There are many causes of cirrhosis, hepatitis, alcoholism, nonalcoholic fatty liver disease, and Budd-Chiari syndrome [3, 4]. Further aggravation of the degree of cirrhosis results in a variety of complications, such as abnormal liver function, portal hypertension, gastroesophageal varices, hypersplenism, ascites, hepatic encephalopathy, and hepatorenal syndrome [5–7]. Hypersplenism is defined as a clinical syndrome characterized by pathological enlargement of the spleen, cytopenias, and compensatory hyperplasia of the bone marrow, and the main clinical manifestations are anemia, infection, bleeding, and decreased immune function [8]. 70–80% of patients with decompensated cirrhosis have hypersplenism [9]. The main treatments for hypersplenism include medical therapy, surgery, and interventional therapy [10]. Among the interventional treatments, partial splenic embolization (PSE) is the most commonly used, and it was first reported in 1973 [11]. Due to the poor long-term effect of medical treatment and the high risk and complications of surgical treatment, more and more patients choose partial splenic embolization. Partial splenic embolization can not only effectively increase the number of peripheral blood cells in patients with cirrhosis, but also reduce portal venous pressure [12] and reduce the risk of gastroesophageal variceal bleeding [13]. Studies have reported that the efficacy of partial splenic embolization is positively correlated with the volume of splenic embolization. However, the larger the volume of splenic embolization, the higher the risk of postoperative complications [14]. Common complications after partial splenic embolization are post-embolization syndrome, portal vein thrombosis, pleural effusion, ascites, peritonitis, splenic abscess, and acute respiratory distress syndrome [15]. Massive splenomegaly is defined as the lower edge of the spleen beyond the level of the umbilicus, or the ventral midline [16]. Studies have reported that complications after PSE often occur in patients whose embolic volume exceeds 50% [17]. For patients with massive splenomegaly, with the same spleen infarction rate, the actual volume of spleen infarction is larger, and the risk of complications is higher. The aim of this study was to investigate the efficacy and safety of the combination of low-molecular-weight heparin + dexamethasone after partial splenic embolization in cirrhotic patients with massive splenomegaly, and whether it can reduce the occurrence of complications without affecting the efficacy.

## Materials and methods

### General information

The data of 116 patients with liver cirrhosis complicated with massive splenomegaly who underwent partial splenic embolization in the Department of Intervention, Union Hospital, Tongji Medical College, Huazhong University of Science and Technology from January 2016 to December 2019 were collected. Inclusion criteria (1) Patients with liver cirrhosis and portal hypertension diagnosed by imaging and (or) endoscopy, and also meet the diagnostic criteria of hypersplenism; (2) The lower edge of the spleen is beyond the level of the umbilicus, or the midline of the abdomen; (3) Aged 18–70 years old; (4) Liver function classification: Child–Pugh A-B, performance score (ECOG) 0–2 points; (5) No portal vein thrombosis and vascular malformations; (6) No gastrointestinal bleeding; (7) Complete clinical follow-up data. Exclusion criteria: (1) Liver function classification: Child–Pugh C, physical score (ECOG) > 2 points; (2) combined with other vital organ dysfunction, such as heart, lung and renal insufficiency; (3) severe bleeding tendency or coagulation dysfunction; (4) previously received blood transfusion, leukocyte-elevating, platelet-elevating and other treatments; (4) combined with malignant tumors; (5) patients who underwent transjugular intrahepatic portosystemic shunt (TIPS); (6) combined with other blood diseases causing splenomegaly, hypersplenism; (7) ascites; (8) allergic to drugs used in the treatment program. Patients were divided into two groups according to whether they received combined treatment with low-molecular-weight heparin + dexamethasone after partial splenic embolization (PSE): PSE + heparin + dexamethasone group (N = 54) and PSE group (N = 62). The baseline data of patients in the two groups were collected, including: gender, age, etiology of liver cirrhosis, preoperative Child–Pugh classification of liver function, ECOG score, total bilirubin, albumin, BUN, creatinine, white blood cells, red blood cells, and platelets.

### Method

#### PSE process

The patient was positioned supine, disinfected in the inguinal region, and draped aseptically. Local anesthesia was performed at the puncture site using 2% lidocaine, the femoral artery was punctured using the Seldinger technique, and a 5F catheter sheath was placed. 5F Yashino catheter was used for cannulation to the celiac axis and splenic artery for angiography. After the course of splenic artery was confirmed, the catheter was cannulated to the end of splenic artery trunk. Appropriate amount of PVA particles with particle size of 300–-500 um + contrast agent suspension was slowly injected for embolization. The angiography reexamination assessed that the splenic embolization volume reached 40–50%. At the end of the treatment, the catheter was removed and the puncture site was pressurized and dressed.

The incidence of postoperative adverse reactions was observed using the Common Terminology Criteria for Adverse Events (CTCAE 4.0). Patients were monitored for the occurrence of abdominal pain within 10 days after PSE, which was assessed using the VAS visual analogue scale. The volume of spleen was measured before operation and 1 month after operation. The volume of splenic embolism area and embolization rate were calculated. Spleen volume was measured using volume software within Siemens CT workstation, which was automatically calculated by the software after manual labeling of the spleen layer by layer. Imaging examination was reexamined at 1 month after operation; blood routine, liver and kidney function and other indicators were reexamined at 1, 3, 6, 9 and 12 months after operation.

#### Treatment with low molecular weight heparin + dexamethasone

Low-molecular-weight heparin was administered at 4000 IU subcutaneously once daily for 1 week starting on the day of PSE. Dexamethasone administration: 5 mg, IV bolus, every other day for 5 doses starting on the day of PSE.

### Outcome measures

1. The volume of spleen, the volume of spleen embolism and the embolization rate of spleen in the two groups at 1 month after PSE;
2. The changes of peripheral blood cells in the two groups during the follow-up period after treatment;
3. Occurrence of treatment-related adverse events after PSE in the two groups;
4. The degree of postoperative abdominal pain in the two groups;
5. The changes of liver and kidney function and physical status before and after treatment in the two groups;

### Statistical methods

Statistical analysis was performed using SPSS software (Version24.0, IBM, Armonk, NewYork). Measurement data were expressed as mean ± standard deviation, and differences were compared using the t-test. Number of cases (percentage) was used for enumeration data, and chi-square test was used for comparison of differences, including Pearson Chi-Square and Fisher Exact Test. Differences were considered statistically significant if the *P* value was < 0.05.

## Results

### Comparison of baseline data between the two groups

The age was 51.4 ± 9.3 years in the PSE + Hep + DEX group and 50.3 ± 10.2 years in the PSE group (*P* = 0.586). The causes of cirrhosis in the PSE + Hep + DEX group were: 34 patients (62.9%) with hepatitis B; 10 patients (18.5%) with hepatitis C; 9 patients (16.7%) with alcoholic cirrhosis; and 1 patient (1.9%) with autoimmune hepatitis. The causes of cirrhosis in the PSE group were: 33 patients (53.2%) with hepatitis B; 19 patients (30.7%) with hepatitis C; and 10 patients (16.1%) with alcoholic cirrhosis. The enumeration data of patients in the two groups were compared using the chi-square test. There was no statistically significant difference in gender, etiology of cirrhosis, pretreatment ECOG score and liver function grade between the two groups (*P* > 0.05, Shown in Table 1).

**Table 1:** Comparison of baseline data before PSE between the two groups

			Group		Chi-square tests (*P* value)	t-test (*P* value)
			PSE + Hep + DEX group (N = 54)	PSE group (N = 62)		
Gender	Female	Count (%)	11 (20.4%)	11 (17.7%)	0.814	
	Male	Count (%)	43 (79.6%)	51 (82.3%)		
Etiology of cirrhosis	Hepatitis B	Count (%)	34 (62.9%)	33 (53.2%)	0.344	
	Hepatitis C	Count (%)	10 (18.5%)	19 (30.7%)		
	Alcoholic cirrhosis	Count (%)	9 (16.7%)	10 (16.1%)		
	Autoimmune cirrhosis	Count (%)	1 (1.9%)	0 (0.0%)		
Pre-treatment ECOG	0	Count (%)	22 (40.7%)	26 (41.9%)	0.528	
	1	Count (%)	26 (48.2%)	25 (40.3%)		
	2	Count (%)	6 (11.1%)	11 (17.8%)		
Pre-treatment liver function	Child A	Count (%)	35 (64.8%)	42 (67.7%)	0.739	
	Child B	Count (%)	19 (35.2%)	20 (32.3%)		
Age (Years)	Mean ± SD		51.4 ± 9.3	50.3 ± 10.2		0.586
Pre-treatment bilirubin (μmol/L)	Mean ± SD		14.0 ± 5.1	13.9 ± 6.2		0.919
Pretreatment Albumin (g/L)	Mean ± SD		35.27 ± 3.39	34.29 ± 3.64		0.139
Pretreatment BUN (mmol/L)	Mean ± SD		5.67 ± 1.72	5.51 ± 1.47		0.608
Pretreatment Cr (μmol/L)	Mean ± SD		74.1 ± 17.6	77.7 ± 19.8		0.308
Pretreatment WBC (G/L)	Mean ± SD		1.98 ± 0.41	2.05 ± 0.56		0.491
Pretreatment RBC (T/L)	Mean ± SD		3.08 ± 0.57	2.97 ± 0.55		0.323
Pretreatment PLT (G/L)	Mean ± SD		32.74 ± 11.93	35.26 ± 12.49		0.271

Tests of normality		
	Group	Kolmogorov–Smirnov^a^
		*P* value
Age (Years)	PSE + Hep + DEX group	0.116
	PSE group	0.131
Pre-treatment bilirubin (μmol/L)	PSE + Hep + DEX group	0.356
	PSE group	0.452
Pretreatment Albumin (g/L)	PSE + Hep + DEX group	0.414
	PSE group	0.607
Pretreatment BUN (mmol/L)	PSE + Hep + DEX group	0.513
	PSE group	0.767
Pretreatment Cr (μmol/L)	PSE + Hep + DEX group	0.247
	PSE group	0.171
Pretreatment WBC (G/L)	PSE + Hep + DEX group	0.091
	PSE group	0.113
Pretreatment RBC (T/L)	PSE + Hep + DEX group	0.138
	PSE group	0.073
Pretreatment PLT (G/L)	PSE + Hep + DEX group	0.120
	PSE group	0.077

^a^Lilliefors significance correction

The comparison of total bilirubin, albumin, BUN, Cr, white blood cells, red blood cells and platelets before treatment between the two groups was performed using t-test, *P* value > 0.05, without statistical difference (Shown in Table 1).

### Comparison of liver and kidney function, liver function classification and physical status between the two groups at 1 month after treatment

There was no significant difference in ECOG score (*P* = 0.260) and liver function grade (*P* = 0.937) between the two groups after treatment (*P* > 0.05). After treatment, total bilirubin (*P* = 0.163), albumin (*P* = 0.139), BUN (*P* = 0.512) and Cr (*P* = 0.309) in the two groups were compared using T test, *P* > 0.05, without statistical difference (Shown in Table 2).

**Table 2:** Comparison of liver function, renal function and performance status after PSE between the two groups

			Group		Chi-square tests (*P* value)	t-test (*P* value)
			PSE + Hep + DEX group (N = 54)	PSE group (N = 62)		
Post-Treatment ECOG	0	Count (%)	19 (35.2%)	29 (46.8%)	0.260	
	1	Count (%)	30 (55.6%)	25 (40.3%)		
	2	Count (%)	5 (9.2%)	8 (12.9%)		
Post-treatment liver function	Child A	Count (%)	43 (79.6%)	49 (79.0%)	0.937	
	Child B	Count (%)	11 (20.4%)	13 (21.0%)		
Post-treatment bilirubin (μmol/L)	Mean ± SD		15.2 ± 5.1	13.8 ± 5.5		0.163
Post-treatment Albumin (g/L)	Mean ± SD		34.42 ± 3.06	33.58 ± 2.99		0.139
Post-treatment BUN (mmol/L)	Mean ± SD		5.96 ± 1.48	6.15 ± 1.49		0.512
Post-Treatment Cr (μmol/L)	Mean ± SD		72.2 ± 19.5	76.1 ± 20.9		0.309

Tests of Normality		
	Group	Kolmogorov–Smirnov^a^
		*P* value
Post-treatment bilirubin (μmol/L)	PSE + Hep + DEX group	0.218
	PSE group	0.201
Post-treatment Albumin (g/L)	PSE + Hep + DEX group	0.132
	PSE group	0.097
Post-treatment BUN (mmol/L)	PSE + Hep + DEX group	0.324
	PSE group	0.166
Post-Treatment Cr (μmol/L)	PSE + Hep + DEX group	0.253
	PSE group	0.107

^a^Lilliefors significance correction

### Compare the changes of liver and kidney function 1 month after treatment in each group

T-test was used to compare total bilirubin (*P* = 0.265), albumin (*P* = 0.275), BUN (*P* = 0.157) and Cr (*P* = 0.459) before and after treatment in PSE + Hep + DEX group, *P* > 0.05, without statistical difference (Shown in Table 3). T-test was used to compare total bilirubin (*P* = 0.959), albumin (*P* = 0.235) and Cr (*P* = 0.541) before and after PSE group treatment, *P* > 0.05, without statistical difference (see Table 3). In PSE group, BUN was 5.51 ± 1.47 mmol/L before treatment and 6.15 ± 1.49 mmol/L after treatment, and BUN increased after treatment compared with that before treatment (*P* = 0.001), and the difference had statistical significance (Shown in Table 3).

**Table 3:** Comparison of liver and kidney function before and after PSE in each group

Group			Mean ± SD	t-test (*P* value)
PSE + Hep + DEX group (N = 54)	Bilirubin (μmol/L)	Before treatment	14.0 ± 5.1	0.265
		Post Treatment	15.2 ± 5.1	
	Albumin (g/L)	Before treatment	35.27 ± 3.39	0.275
		Post Treatment	34.42 ± 3.06	
	BUN (mmol/L)	Before treatment	5.67 ± 1.72	0.157
		Post Treatment	5.96 ± 1.48	
	Cr (μmol/L)	Before treatment	74.1 ± 17.6	0.459
		Post Treatment	72.2 ± 19.5	
PSE group (N = 62)	Bilirubin (μmol/L)	Before treatment	13.9 ± 6.2	0.959
		Post Treatment	13.8 ± 5.5	
	Albumin (g/L)	Before treatment	34.29 ± 3.64	0.235
		Post Treatment	33.58 ± 2.99	
	BUN (mmol/L)	Before treatment	5.51 ± 1.47	0.001
		Post Treatment	6.15 ± 1.49	
	Cr (μmol/L)	Before treatment	77.7 ± 19.8	0.541
		Post Treatment	76.1 ± 20.9	

### Comparison of spleen volume before treatment, spleen volume after treatment, volume of splenic embolism and splenic embolism rate between the two groups

**Fig. 1:**
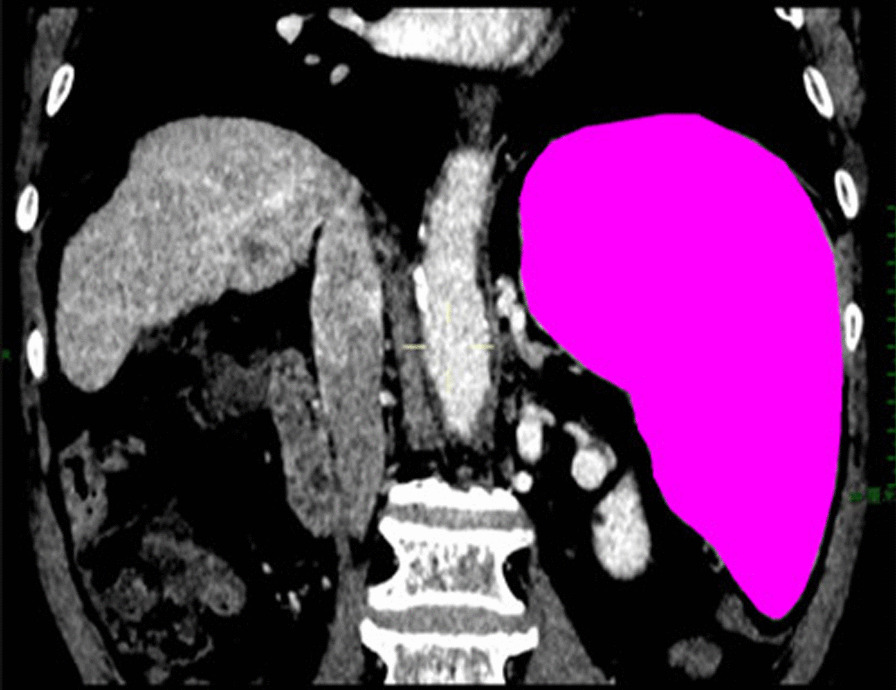
Before partial splenic embolization, the spleen volume was 1786.79 cm^3^

**Fig. 2:**
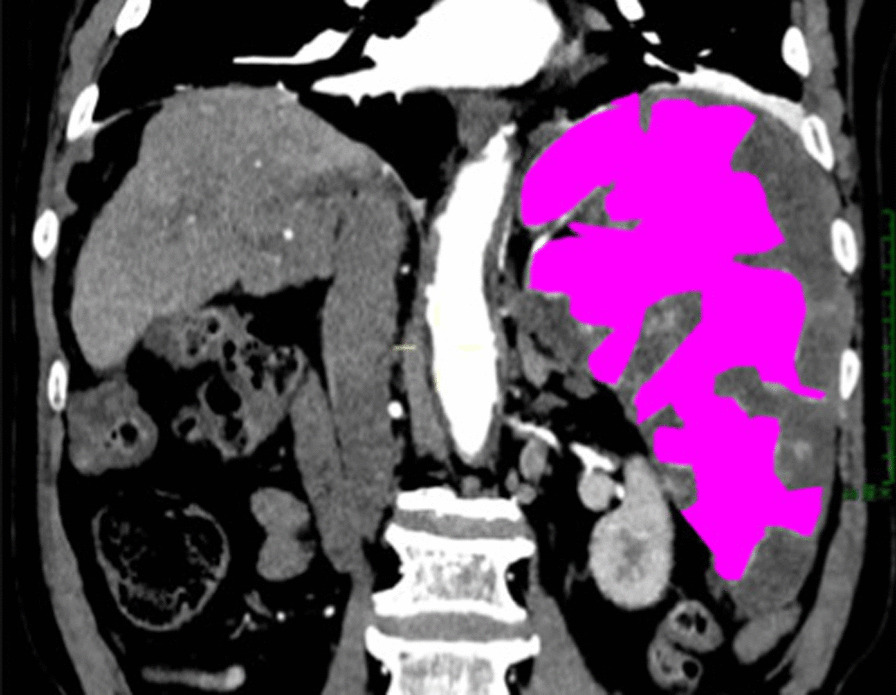
After partial splenic embolization, the spleen volume was 1023.24 cm^3^

The spleen volume before treatment was compared between the two groups, *P* = 0.385. The volume of spleen was compared between the two groups at 1 month after treatment, *P* = 0.417. The volume of splenic embolization was 622.34 ± 157.06 cm^3^ in the PSE + Hep + DEX group and 587.62 ± 175.33 cm^3^ in the PSE group (*P* = 0.306). The embolization rate of spleen was compared between the two groups, *P* = 0.573. Shown in Table 4, t-test was used for comparison between two groups, *P* > 0.05, without statistical difference. Typical cases are shown in Figs. 1 and 2.

**Table 4:** Comparison of preoperative spleen volume, postoperative spleen volume, spleen embolization volume and spleen embolization rate between the two groups

		Group		t-test (*P* value)
		PSE + Hep + DEX group (N = 54)	PSE group (N = 62)	
Preoperative spleen volume (cm^3^)	Mean ± SD	1386.54 ± 429.62	1253.73 ± 547.28	0.385
Postoperative spleen volume (cm^3^)	Mean ± SD	715.19 ± 352.43	698.47 ± 371.81	0.417
Spleen embolization volume (cm^3^)	Mean ± SD	622.34 ± 157.06	587.62 ± 175.33	0.306
Spleen embolization rate (%)	Mean ± SD	47.3 ± 13.6	45.8 ± 17.1	0.573

### Comparison of WBC and PLT at different follow-up stages after treatment between the two groups

WBC increased significantly after treatment compared with that before treatment in both groups. At 1 week after operation, WBC in PSE + Hep + DEX group was 9.13 ± 4.45 G/L, WBC in PSE group was 7.42 ± 3.61 G/L; at 1 week, PSE + Hep + DEX group had higher WBC value, *P* = 0.027 for the comparison between the two groups, with statistical significance. At a later follow-up stages, WBC showed a gradually decreasing trend in both groups. The number of WBC at 1 month, 3 months, 6 months, 9 months and 12 months after treatment was compared between the two groups, *P* > 0.05, without statistical difference.

PLT was significantly increased after treatment compared with that before treatment in both groups. The number of PLT in the two groups peaked at 1 month after treatment. PSE + Hep + DEX group was 131.5 ± 23.6 G/L and PSE group was 119.3 ± 21.5 G/L. From 1 month after treatment, the number of PLT in the two groups showed a gradually decreasing trend. At 12 months after treatment, PLT was 103.3 ± 24.9G/L in the PSE + Hep + DEX group and 102.5 ± 17.9G/L in the PSE group. The number of PLT in the PSE + Hep + DEX group was higher than that in the PSE group at 1 week and 1 month after treatment (*P* = 0.014, *P* = 0.035). The number of PLT at 3 months, 6 months, 9 months and 12 months after treatment was compared between the two groups, *P* > 0.05, without statistical difference.

Shown in Table 5, t-test for comparison between two groups. Statistical differences were accepted if *P* < 0.05.

**Table 5:** Comparison of WBC and PLT at different stages after PSE between the two groups

		Group		t-test (*P* value)
		PSE + Hep + DEX group (N = 54)	PSE group (N = 62)	
1 week	WBC (G/L)	9.13 ± 4.45	7.42 ± 3.61	0.027
	PLT (G/L)	103.2 ± 28.7	86.4 ± 19.2	0.014
1 month	WBC (G/L)	5.46 ± 2.75	5.09 ± 3.12	0.103
	PLT (G/L)	131.5 ± 23.6	119.3 ± 21.5	0.035
3 months	WBC (G/L)	4.71 ± 1.36	4.83 ± 1.43	0.672
	PLT (G/L)	124.6 ± 22.0	112.5 ± 24.7	0.326
6 months	WBC (G/L)	4.15 ± 1.17	4.01 ± 1.06	0.491
	PLT (G/L)	113.1 ± 26.5	109.3 ± 23.4	0.579
9 months	WBC (G/L)	3.95 ± 0.84	3.81 ± 1.12	0.388
	PLT (G/L)	105.7 ± 19.4	101.4 ± 21.3	0.502
12 months	WBC (G/L)	3.80 ± 1.23	3.76 ± 0.97	0.463
	PLT (G/L)	103.3 ± 24.9	102.5 ± 17.9	0.617

### Comparison of adverse events (AEs) after treatment between the two groups

The incidence of abdominal pain was lower in the PSE + Hep + DEX group than in the PSE group (46.3% vs 66.1%, *P* = 0.039). The incidence of fever was lower in the PSE + Hep + DEX group than in the PSE group (38.9% vs 75.8%, *P* < 0.001). The incidence of portal vein thrombosis (PVT) was lower in the PSE + Hep + DEX group than in the PSE group (1.9% vs 12.9%, *P* = 0.026). The incidence of refractory ascites was lower in the PSE + Hep + DEX group than in the PSE group (5.6% vs 19.4%, *P* = 0.027). The difference in the incidence rate of vomiting, pleural effusion and ascites between the two groups had no statistical significance (*P* > 0.05). Splenic abscess occurred in 1(1.6%) patient in the PSE group and none (0.0%) in the PSE + Hep + DEX group (*P* = 0.349).

Shown in Table 6, Chi-square test was used for comparison between two groups. Statistical differences were accepted if *P* < 0.05.

**Table 6:** Incidence of adverse events after treatment in the two groups

			Group		Chi-square test (*P* value)
			PSE + Hep + DEX group (N = 54)	PSE group (N = 62)	
Abdominal pain	No	Count (%)	29 (53.7%)	21 (33.9%)	0.039
	Yes	Count (%)	25 (46.3%)	41 (66.1%)	
Fever	No	Count (%)	33 (61.1%)	15 (24.2%)	0.000
	Yes	Count (%)	21 (38.9%)	47 (75.8%)	
Vomiting	No	Count (%)	46 (85.2%)	47 (75.8%)	0.248
	Yes	Count (%)	8 (14.8%)	15 (24.2%)	
Hydrothorax	No	Count (%)	45 (83.3%)	43 (69.4%)	0.079
	Yes	Count (%)	9 (16.7%)	19 (30.6%)	
Ascites	No	Count (%)	41 (75.9%)	38 (61.3%)	0.092
	Yes	Count (%)	13 (24.1%)	24 (38.7%)	
Portal vein thrombosis	No	Count (%)	53 (98.1%)	54 (87.1%)	0.026
	Yes	Count (%)	1 (1.9%)	8 (12.9%)	
Splenic abscess	No	Count (%)	54 (100.0%)	61 (98.4%)	0.349
	Yes	Count (%)	0 (0.0%)	1 (1.6%)	
Refractory ascites	No	Count (%)	51 (94.4%)	50 (80.6%)	0.027
	Yes	Count (%)	3 (5.6%)	12 (19.4%)	

### Comparison of VAS score of abdominal pain after PSE between the two groups

Abdominal pain after PSE peaked on postoperative days 4–5 in both groups of patients, followed by a gradual decrease. The VAS score of abdominal pain in PSE group was significantly higher than that in PSE + Hep + DEX group on postoperative days 2–8, and the *P* < 0.05 for the comparison between the two groups. Among them, the PSE group achieved a VAS score of 8.3 ± 2.2 for abdominal pain on the fifth postoperative day. On the 9th to 10th day after PSE, the VAS score of abdominal pain in the two groups was significantly lower, and there was no statistically significant difference between the two groups (*P* > 0.05).

Shown in Table 7, t-test for comparison between two groups. Statistical differences were accepted if *P* < 0.05.

**Table 7:** Comparison of VAS score of abdominal pain after PSE between the two groups

	Group		t-test (*P* value)
	PSE + Hep + DEX group (N = 54)	PSE group (N = 62)	
Day 1	2.7 ± 2.2	2.9 ± 1.4	0.337
Day 2	3.5 ± 2.4	4.6 ± 2.3	0.041
Day 3	4.3 ± 1.9	5.9 ± 2.1	0.032
Day 4	5.8 ± 2.7	7.6 ± 2.7	0.015
Day 5	5.2 ± 1.3	8.3 ± 2.2	0.006
Day 6	4.1 ± 1.5	7.2 ± 1.9	0.010
Day 7	3.2 ± 0.9	5.5 ± 1.3	0.024
Day 8	2.4 ± 1.1	4.3 ± 1.6	0.048
Day 9	1.8 ± 1.0	2.1 ± 0.8	0.306
Day 10	1.7 ± 1.2	1.9 ± 0.9	0.579

## Discussion

Most scholars believe that the pathogenesis of hypersplenism is that after the enlargement of the spleen, a large number of blood cells remain in the spleen [18], which activates the phagocytic system in the spleen, destroys the blood cells deposited in the spleen, resulting in the decrease of blood cell count. Liver cirrhosis combined with hypersplenism will also lead to blood flow redistribution. The thickened splenic artery will lead to a large amount of arterial blood flowing into the spleen, even up to 19% of cardiac output, resulting in splenic artery steal syndrome [19]. Splenectomy is one of the methods to treat hypersplenism, but the incidence and severity of its complications are high. Splenectomy may lead to portal vein thrombosis, affect the patient's liver function and increase the risk of gastrointestinal bleeding [20]. After splenectomy, the levels of *P* factor, opsonin and immune factor tuftsin in peripheral blood decreased, and the immune function of the body decreased. Bacteria can rapidly multiply in a short time, resulting in overwhelming post-splenectomy infection (OPSI) syndrome [21]. In addition, the loss of spleen tissue can induce the formation of pulmonary hypertension [22], which also increases the incidence and mortality of pneumonia and heart disease [23]. Partial splenic embolization (PSE) is a minimally invasive, safe and effective treatment for hypersplenism. The theoretical basis of PSE is derived from the anatomical structure of the spleen [24, 25]. The spleen parenchyma is divided into red pulp, white pulp and marginal area. The white pulp, which is located in the center and composed of dense lymphoid tissue, is the main site of specific immunity. The red pulp is located in the periphery of the spleen and is composed of splenic cord and blood sinus. When the spleen is hyperfunctional, a large number of blood cells stagnate in the red pulp. During partial splenic embolization, the embolic site is the branch below the central artery, which mainly reduces the volume of the red pulp and thus improves the peripheral blood cells, without affecting the immune function of the spleen [23]. Jin et al. [26] reported that although PSE may affect the immune function of patients with cirrhosis and hypersplenism in a short time, the immune function of patients will gradually return to normal after PSE. Research reported [27] that WBC and PLT began to rise 1–3 days after operation, showing a gradual upward trend. WBC increased in a short time, which may also be related to the inflammatory reaction caused by splenic necrosis. The results of this study showed that WBC and PLT of the two groups increased significantly and reached the peak 1 month after treatment. Although WBC and PLT decreased during continued follow-up, WBC and PLT increased significantly in both groups at 1 year of follow-up compared with those before treatment, maintaining a safe level. Nio et al. [28] reported that platelets began to increase from 12 to 24 h after PSE and peaked at 1–2 weeks; platelet counts would remain stable within 1–2 months and then slowly decrease, but still significantly higher than before treatment. Tajiri et al. [29] reported that platelets peaked after 2 weeks after PSE, and then although there was a slow decrease, platelet counts increased significantly for up to 8 years compared with those before PSE. It is reported by Tan et al. [30] that platelets began to rise 1 week after PSE and peaked 1 month after treatment. Zaitoun et al. [31] reported that PLT reached a peak level of 155.56 ± 30.7 G/L and WBC rose to a peak level of 7.5 ± 1.7 G/L 2 weeks after PSE. It is reported by DuBois et al. [32] that PSE is efficacious in increasing WBC count out to 2 years and platelet count out to 3.5 years in patientswith hypersplenism. Gu et al. [33] reported that WBC peaked at 2 weeks after PSE and then gradually decreased, but it was still significantly higher than the preoperative level during the 4 years of follow-up. The results of this study showed that the higher WBC values in the PSE + Hep + DEX group at 1 week may be related to the use of dexamethasone. This study found that the PLT in the PSE + Hep + DEX group was higher than that in the PSE group at 1 week and 1 month after treatment. To analyze the possible reasons, in addition to the use of dexamethasone, it was also associated with a lower incidence of postoperative portal vein thrombosis and less platelet consumption in the PSE + Hep + DEX group.

Common complications after partial splenic embolization are post- embolization syndrome (such as abdominal pain, fever, nausea and vomiting) [34], portal vein thrombosis, hydrothorax, ascites and splenic abscess [35]. Studies have reported [36] that the volume of the embolized spleen is associated with efficacy and the occurrence of complications. Although the larger the volume of splenic embolization, the better the curative effect, the higher the risk of complications [37]. The smaller the volume of splenic embolization, the lower the risk of complications, but the curative effect is often poor. Multiple studies have evaluated the relationship between the degree of splenic embolization and the safety of PSE, and it is agreed that the degree of embolization is positively correlated with complications [38]. Studies have reported [37, 39] that embolization of 25–40% can effectively improve peripheral hemogram and preserve splenic immune function; embolization of 60–80% can reduce portal venous pressure and reduce the risk of upper gastrointestinal bleeding; and the incidence of serious complications is significantly increased after embolization of more than 70% [14]. Lee et al. [40] reported that the extent of embolization in the spleen of PSE was more than 30% to effectively increase platelets. According to the research of Mukaiya et al. [41], the patients were divided into three groups according to the scope of splenic embolism: < 50%, 50%-70% and ≥ 70%, and the incidence of postoperative complications was 28%, 56% and 95% respectively.

In patients with massive splenomegaly, the actual volume of spleen is very large. Under the same proportion of embolization, the absolute volume of spleen necrosis is larger, and the risk of postoperative complications is higher. The most common complication after PSE is post embolism syndrome [42], which is related to the absorption of necrotic substances and aseptic inflammatory reaction after spleen infarction. The greater the absolute volume of splenic infarction, the higher the probability and severity of post embolic syndrome, affecting the postoperative recovery of patients, prolonging the hospitalization time of patients, and reducing the treatment compliance of patients. Portal vein thrombosis (PVT) is one of the most serious complications after PSE. The cause of PVT is that after PSE, the blood flow and velocity of splenic vein decrease significantly, which makes the blood flow velocity of portal vein slow down significantly, and even vortex appears. It is reported that the larger the spleen and the wider the diameter of splenic vein, the more obvious the decrease of flow velocity after PSE, and the easier it is to form portal vein thrombosis. Ogawa et al. [43] reported that the diameter of splenic vein and the degree of splenic embolism are independent risk factors for portal vein thrombosis in PSE. After PSE, the destruction of spleen to blood cells decreased, WBC and PLT in peripheral blood increased significantly, and blood viscosity increased; PLT in peripheral blood was significantly increased, and the patient's blood was in hypercoagulable state; After PSE, the levels of anticoagulation related protein S and protein C decreased, and the level of antithrombin III decreased, which increased the risk of thrombosis. PVT can not be relieved automatically after its formation. Once the treatment opportunity is missed, it will bring serious consequences and increase the mortality of cirrhotic patients with portal hypertension. Therefore, in this study we used low-molecular-weight heparin and dexamethasone to reduce the incidence of the most common and dangerous complications after PSE in patients with liver cirrhosis and megasplenomegaly. Low molecular weight heparin can bind to antithrombin III, resulting in structural changes of antithrombin III, thereby accelerating the inhibitory effect on factor Xa, producing anticoagulant effect, and having less effect on antithrombin, so it can reduce heparin-induced bleeding and other adverse reactions while achieving effective anticoagulant effect [44]. Dexamethasone is a commonly used steroid hormone with the effects of immunosuppression, anti-endotoxin and enhancing the body's stress response [45]. It can inhibit the accumulation of inflammatory cells at the site of inflammation, and inhibit phagocytosis, release of lysosomal enzymes, and synthesis and release of inflammatory mediators, thereby reducing the tissue response to inflammation. Dexamethasone can prevent or inhibit cell-mediated immune responses and has immunosuppressive effects. Studies have reported that dexamethasone can maintain the integrity of lysosomal membranes and regulate vascular permeability by strengthening cell–cell contact. Dexamethasone plays an important role in regulating the inflammatory response due to its strong role in stabilizing the endothelium. The results of this study found that the incidence of postoperative abdominal pain (46.3% vs 66.1%) and fever (38.9% vs 75.8%) was reduced in the PSE + Hep + DEX group compared with the PSE group, with statistically significant differences (*P* < 0.05). Moreover, on postoperative d2-8 after PSE, the VAS score of abdominal pain severity in PSE + Hep + DEX group was lower than that in PSE group, and the difference had statistical significance (*P* < 0.05). The reason is that dexamethasone has a strong anti-inflammatory effect, which reduces the incidence of aseptic inflammation after PSE and reduces the degree of inflammatory reaction. Yu et al. [46] reported that the duration of fever was 3.36 ± 2.31 days and the duration of pain was 7.39 ± 4.00 days in the dexamethasone group, and dexamethasone was effective in relieving post-embolization syndrome after PSE in patients. There are not many reports on dexamethasone for prevention of post-embolization syndrome in PSE. However, there are many reports of dexamethasone in preventing post-embolization syndrome after transcatheter arterial chemoembolization (TACE), and the mechanism of post-embolization syndrome after TACE is similar to that of PSE. It is reported by Sainamthip et al. [47] that the use of dexamethasone was effective in preventing the occurrence of post-embolization syndrome after TACE in hepatocellular carcinoma patients.

Portal vein thrombosis is mostly lack of specific clinical symptoms. At the initial stage of onset, it is often manifested as abdominal pain, fever, nausea, vomiting or increased ascites, which is not easy to distinguish from post embolism syndrome; Severe cases may occur upper gastrointestinal bleeding, liver failure, intestinal necrosis, etc.; Once the thrombus is formed, it is often organized rapidly. At this time, the best time for treatment has been missed, resulting in the poor effect of drug thrombolysis. Thrombosis also greatly increases the mortality of patients with portal hypertension. Brandt et al. [48] reported that 4 of 17 patients with hypersplenism had portal vein thrombosis after PSE. It is reported by Matsumoto et al. [49] that 8 (50%) of 16 patients with PSE had portal vein or splenic vein thrombosis by multidetector row CT (MDCT). The results of this study showed that the incidence of portal vein thrombosis (1.9% vs 12.9%) and refractory ascites (5.6% vs 19.4%) in PSE + Hep + Dex group was lower than that in PSE group (*P* < 0.05). The low incidence of portal vein thrombosis was attributed to the effect of low molecular weight heparin. Amin et al. [50] reported that one of the 20 patients with PSE developed portal vein thrombosis and improved after anticoagulation treatment. It is reported by Cai et al. [51] that 145 patients with liver cirrhosis and hypersplenism underwent PSE, 11 patients developed portal vein thrombosis, and 5 patients received anticoagulation therapy. Thrombus disappeared in 4 of the 5 cases after treatment, and there was no progress in 1 case. Among the 6 patients who did not receive anticoagulant therapy, 2 patients developed gastrointestinal hemorrhage due to thrombosis, 3 patients developed cavernous transformation of portal vein with aggravation of varicose veins, and 1 patient developed thrombus calcification. Therefore, he suggested that early detection of portal vein thrombosis and early anticoagulation after PSE could effectively avoid serious complications. N'Kontchou et al. [52] reported that 2 of 32 patients receiving PSE developed PVT, and the thrombus disappeared after anticoagulation treatment. It is reported by Wu et al. [53] that postoperative prophylactic antithrombotic therapy is a protective factor for portal vein thrombosis after PSE. Refractory ascites refers to that there is no response to dietary sodium restriction (< 90 mmol/l) and high-dose diuretics (spironolactone 400 mg/day and furosemide 160 mg/day) for at least 1 week, or serious electrolyte disorder and hepatic encephalopathy occur during the use of diuretics. Some scholars have reported that refractory ascites is one of the clinical manifestations of portal vein thrombosis in PSE. The results of this study showed that the incidence of refractory ascites in PSE + Hep + Dex group was lower than that in PSE group. On the one hand, the reason may be that the degree of aseptic inflammation after dexamethasone use was less, on the other hand, the portal vein blood flow was more unobstructed after low molecular weight heparin use. The results of this study showed that there was no significant difference in the incidence of splenic abscess between PSE + Hep + Dex group and PSE group (0.0% vs 1.6%, *P* < 0.05), indicating that the use of dexamethasone is safe, which is consistent with the results of other studies. Yu et al. [46] reported that 30 patients with PSE were treated with dexamethasone to prevent post embolism syndrome, and there was no splenic abscess. In this study, no bleeding and other complications occurred in both groups, indicating that the combined use of low molecular weight heparin after PSE is safe, which is also consistent with the results of Cai [51].

The results of this study showed that there was no significant difference in liver function before and after treatment between the two groups (*P* > 0.05). Numata et al. [54] reported that PSE was also effective in improving liver function status in cirrhotic patients with hypersplenism. It is found by Pang et al. [55] that total bilirubin levels decreased in patients after PSE and returned to normal levels after 6 months. Hayashi et al. [56] found that 1 year after PSE, patients' serum albumin and cholinesterase increased to 104 ± 14% and 130 ± 65% of pretreatment levels, respectively. Nomiyama et al. [57] reported that PSE can improve liver function without serious complications in patients with cirrhosis. There was no significant difference in BUN and Cr before and after treatment in PSE + Hep + DEX group (*P* > 0.05). There was no significant difference in Cr before and after treatment in PSE group (*P* > 0.05). BUN was slightly increased after treatment in the PSE group, which was statistically different from BUN before treatment (*P* < 0.05). The reason may be that after partial splenic embolization in PSE group, the inflammatory reaction is heavier, the release of inflammatory mediators is more, and the burden on the kidney is greater, so the renal function changes. Another possibility is that there are more patients with ascites in PSE group, especially more patients with refractory ascites. A large amount of ascites leads to increased abdominal pressure and decreased renal perfusion, leading to changes in renal function.

## Conclusion

Partial splenic embolization (PSE) combined use of dexamethasone and low-molecular-weight heparin can effectively increase the number of peripheral blood cells in patients, which is similar with partial splenic embolization alone. The combined use of dexamethasone and low-molecular-weight heparin after PSE can effectively reduce the incidence and severity of postoperative complications such as post-embolization syndrome and ascites. The incidence of portal vein thrombosis and refractory ascites after PSE was lower in the combination group, but it did not increase the risk of infection or bleeding in patients. Therefore, the combined use of dexamethasone and low-molecular-weight heparin after PSE is a safe and effective treatment strategy that can significantly reduce the incidence of complications after PSE.

The inadequacy of this study is that the data is from a single center, and it is a retrospective study with limited sample size. In the future work, multi-center, large sample, prospective research can be carried out, and a control study can be designed to compare with the currently commonly used splenic embolization materials, so as to provide more help for clinical work.

## Data Availability

The datasets generated and analysed during the current study are available from the corresponding author on reasonable request.
