# Correction: Efficacy and safety of heparin plus dexamethasone after partial splenic embolization for liver cirrhosis with massive splenomegaly

**DOI:** 10.1186/s12876-022-02635-w

**Published:** 2023-01-31

**Authors:** Haohao Lu, Chuansheng Zheng, Bin Xiong, Xiangwen Xia

**Affiliations:** 1 Department of Radiology, Union Hospital, Tongji Medical College, Huazhong University of Science and Technology, Jiefang Avenue #1277, Wuhan, 430022 China; 2 Hubei Province Key Laboratory of Molecular Imaging, Wuhan, 430022 China

## Correction to: BMC Gastroenterology (2022) 22:470 https://doi.org/10.1186/s12876-022-02580-8

After publication of this article [1], the authors reported that affiliation 1 was incorrectly given as ‘Department of Radiology, Wuhan Union Hospital, Tongji Medical College, Huazhong University of Science and Technology, Jiefang Avenue #1277, Wuhan 430022, China’ but should have been 'Department of Radiology, Union Hospital, Tongji Medical College, Huazhong University of Science and Technology, Jiefang Avenue #1277, Wuhan 430022, China’.

The original article [1] has been corrected.
